# RNAi in the cereal weevil *Sitophilus *spp: Systemic gene knockdown in the bacteriome tissue

**DOI:** 10.1186/1472-6750-9-44

**Published:** 2009-05-15

**Authors:** Agnès Vallier, Carole Vincent-Monégat, Anne Laurençon, Abdelaziz Heddi

**Affiliations:** 1 Université de Lyon, INRA, INSA-Lyon, IFR-41, UMR203 BF2I, Biologie Fonctionnelle Insectes et Interactions, 20 ave A Einstein, F-69621 Villeurbanne, France; 2 Université de Lyon, Lyon, F-69003, France, Université Lyon 1, CNRS, UMR5534, Centre de Génétique Moléculaire et Cellulaire, Villeurbanne, F-69622, France

## Abstract

**Background:**

The weevils *Sitophilus *spp. are among the most important cosmopolitan pests of stored cereal grains. However, their biology and physiology are poorly understood, mainly because the insect developmental stages take place within cereal grains and because of the lack of gene specific molecular manipulation.

**Results:**

To gain access to the different insect developmental stages, weevil females were allowed to lay their eggs on starch pellets and hatched embryos were collected by dissolving starch with water. Embryos were transferred between two Glass Plates filled with packed Flour (GPF) to mimic compact texture of the cereal grain, and this system allowed us to recover specific developmental stages. To knockdown the gene expressed in the bacteria-bearing organ (the bacteriome), whole larvae were injected with dsRNA to target the *wpgrp1 *gene and they were then left to develop for a further 4 days period. Quantitative RT-PCR and Western blot analyses on the bacteriome of these animals revealed a down-regulation of the *wpgrp1 *expression, both at transcript and protein levels.

**Conclusion:**

These results demonstrate that whole larval injection with dsRNA results in a high and systemic decrease of both mRNA and protein in the bacteriome tissue. This, along with the possibility of access to the insect developmental stages, opens up a new research avenue for exploring gene specific functions in the cereal weevils.

## Background

Cereals are the world's basic staple food and they provide an energy and protein source for many populations, particularly in the developing world. Unfortunately, cereal grain losses during storage can reach up to 50% of the total harvest in some countries, which represents a world-wide loss equivalent to thousands of millions of euros [1]. Grain weevils *Sitophilus *spp. (Dryophthoridae, Curculionoidea) are well-known as major primary pests of stored cereal grains causing damage and rendering the grain more susceptible to attack by secondary insect pests such as *Tribolium*.

The genus *Sitophilus *includes three cereal feeding species (*Sitophilus oryzae*, *Sitophilus zeamais *and *Sitophilus granarius*). Interestingly, all three species share an intracellular symbiosis with a Gram-negative γ-Proteobacterium [2,3]. Symbiotic bacteria (endosymbionts) are transmitted maternally to the offsprings and, at an early stage of host embryogenesis, these bacteria induce the differentiation of specialized host cells (the bacteriocytes) that house the bacteria, protect them from the host immune system [4,5], and form a symbiotic organ (the bacteriome) that persists throughout the larval stages [6]. Endosymbiotic bacteria balance the insect diet within the cereal grains, which are starch-rich but poor in amino acids, lipids and vitamins [7-9]. This improves mitochondrial energetic metabolism and impacts, thereby, on the insect *fitness*, flight ability and invasive power [10,6,12].

The control of these storage insects is mainly mediated by use of synthetic insecticides, which generate high environmental costs and lead to insecticide resistant strains [13]. Consequently the development of methods facilitating molecular manipulation of these insects is of wide interest. As insect physiology and reproduction are drastically disturbed in the absence of endosymbionts, one innovative strategy would rely on a better understanding of host symbiont interaction.

Sequencing of the *Sitophilus *endosymbiont genome, along with the development of expressed sequence tags helps to the better knowledge of the weevil biology and physiology [14,4,15]. One very promising method for generating targeted down-regulation of gene expression in a wide range of organisms is RNA interference (RNAi) [16]. RNAi also has evolved into a powerful tool for probing gene function in *Drosophila*, *Tribolium*, *Caenorhabditis elegans *and mice. Delivery of dsRNA or siRNA into a cell triggers abrogation of the target mRNA. Previous experiments have shown the feasibility of using RNAi to abrogate the expression of gene transcripts in several insects [17].

A number of animal cells have been shown to naturally take up exogenous dsRNA and use it to initiate RNAi silencing. In some organisms, such as *Drosophila *and *Bombyx mori*, certain cells show an efficient uptake of dsRNA but they seem to be unable to transmit this dsRNA to other cells in the body [17,18]. Organisms, such as *C. elegans*, can both take up dsRNA and spread it systemically to elicit an RNAi response throughout the entire organism. Interestingly, the first systemic RNAi response in insects was documented in the flour beetle *Tribolium castaneum*: an injection of dsRNA into the larvae results in specific gene silencing in adults [19]. This effect can also be transmissible between generations [20]. This coleopteran species is closely related to *Sitophilus *both phylogenetically and ecologically.

We thus undertook research to access the biological and physiological aspects of the *Sitophilus *system based on the use of genomic tools. The weevil lifestyle and behavior limits embryonic, larval and nymphal development to within the grain only. Thus, weevils cannot survive or reproduce on cereal flour in the way that *Tribolium *insects do. Here, we present new rearing conditions that allow experimental access to different embryonic and larval stages of the cereal weevil *Sitophilus*. This system enables us to keep insects alive outside the cereal grains for the whole life cycle. To test the protocols for gene silencing in *Sitophilus *we have selected a gene specifically expressed in the bacteriome, which encodes the peptidoglycan recognition protein (*wpgrp1*) [21,4]. Using injected dsRNA, we present evidence of systemic RNAi in the weevil *Sitophilus *spp.

## Methods

### Insect rearing

Insects from *Sitophilus zeamais *species (Lagoa strain) were reared as described in [21]. Larvae normally grow inside the wheat grains until they emerge as adults one month after the egg laying, at 27.5°C and 70% relative humidity (rh). For RNAi experiments, third-instar larvae were dissected out of the grains and kept alive in a moist atmosphere at 27.5°C for the length of the experiment.

### Using Glass Plates filled with packed Flour (GPF) for artificial rearing

**Figure 1:**
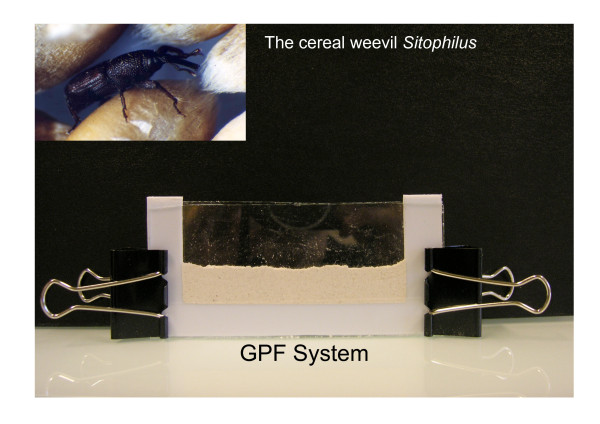
**Glass Plates filled with packed Flour (GPF)**. This artificial system aims to mimic the compactness of the cereal grain. It consists of filling the space between two glass plates, separated with 1.5 mm-thick spacers, with wheat flour. Weevils are individually deposited between two layers of packed wheat flour and transferred to the incubator (27.5°C and 70% relative humidity). This protocol could be adapted to an Eppendorf vial system.

Normally cereal weevil larvae fail to feed outside the grains for behavioral reasons. To overcome this barrier, we have set up an artificial system to mimic the compactness of the cereal grain, which consists of filling with wheat flour the space between two glass plates separated with 1.5 mm-thick spacers (figure 1). Weevil individuals (larvae or embryos) are deposited between two layers of packed wheat flour. We first deposit the first flour layer between the glass plates, then the lavae, and finally we recover the larvae with a second flour layer. This system could also be adapted in an Eppendorf tube.

To access the insect embryonic and post-embryonic stages, both embryos and third-instar larvae were raised within the GPF and kept at 27.5°C and 70% rh until the adults emerged. Females were allowed to lay eggs on starch pellets (L'amidon Remy, ADS) over a period of 4 hours and the embryos were collected by dissolving the starch in water. Embryos were transferred immediately into the GPF system and left in an incubator until the adults emerged. Third-instar larvae were removed directly from the wheat grains after dissection.

### dsRNA synthesis and injection

The method used to synthesize dsRNA is similar to that described by [22]. The primers were designed with the software E-RNAi, available at . For the *wpgrp1 *gene, we used the primers 5'wpgrp1-T7dsRNA and 3'wpgrp1-T7dsRNA that match within the coding sequence, and for the *gfp *gene we used the primers 5'gfp-T7dsRNA and 3'gfp-T7dsRNA (see Table 1). The nucleotides in bold are from the T7 RNA polymerase promoter.

**Table 1:** Primer sequences used for dsRNA synthesis, Reat-time RT PCR and wpgrp1 cDNA amlification. The nucleotide in bold are from the T7 RNA polymerase promoter.

Primers used for dsRNA synthesis	
5'pgrp1-T7dsRNA	5'-TAATACGACTCACTATAGGGCCAGTCCCTTACGTCGTCAT-3'
3'pgrp1-T7dsRNA	5'-TAATACGACTCACTATAGGGTCTGTTTCTCGGACTTGCCT-3'
5'gfp-T7dsRNA 5'	5'-TAATACGACTCACTATAGGGCAAGGAGGACGGCAACATCC-3'
3'gfp-T7dsRNA	5'TAATACGACTCACTATAGGGATTTTATGTTTCAGGTTCAG-3'
	
Primers used for Real-time RT PCR transcript amplification	
wpgrp1-For	5'-ATAATTTCGCTGTTGGAGGG-3'
wpgrp1-Rev	5'-TCTCGGACTTGCCTATGACC-3'
gapdh-For	5'-AACTTTGCCGACAGCCTTGG-3'
gapdh-Rev	5'-GCGCCCATGTATGTAGTTGG-3'
	
Primers uses for PCR amplification of wpgrp1 cDNA	
For-wpgrp1 cDNA	5'-ATGTCCAGTAAGCAATCACGG-3'
Rev-wpgrp1 cDNA	5'-TAACACAATTAGAGTGAAAGATAG-3'

Recombinant plasmids pCR2.1-topo-wpgrp1 [21] and pw8-gfp [23] were used as a matrix to amplify, respectively, *wpgrp1 *and *gfp *with BD Advantage 2 polymerase Mix (BD Biosciences). PCR products were cleaned with a NucleoSpin Extract II kit (MachereyNagel) and were used as templates for *in vitro *dsRNA synthesis using a MEGAscript RNAi Kit (Ambion, Austin, TX). After synthesis, the dsRNA was precipitated overnight at -80°C with 0.3 M sodium acetate, 1.5 μl glycogen and 2 volumes of 100% ethanol, resuspended in water to a final concentration of 2.89 μg/μl. The purity and the integrity were determined by Nanodrop and agarose gel electrophoresis. The RNA was kept at -20°C prior to injection within the following 7 days.

200 ng of dsRNA (69 nl) were injected into the dorsal and posterior part of third-instar larvae using the Nanoinject II nanoinjector (Drummond scientific) and they were kept in the GPF system for 4 days. Bacteriomes were subsequently isolated for RNA extraction.

### Total RNA extraction and cDNA synthesis

Bacteriomes were dissected (25 for each RNA sample) from the fourth instar larvae and total RNA was extracted by RNAqueous^®^-Micro (Ambion), a micro-scale RNA isolation kit, as described in the manufacturers' procedure that includes a DNase treatment final step. After purification, the RNA concentration of each sample was measured by the Nanodrop^® ^spectrophotometer and total RNA quality was checked by electrophoresis. Reverse-transcription into the first strand cDNA was carried out using the First strand Synthesis System for RT-PCR kit (Invitrogen).

### Real-time RT PCR transcript quantification

Quantitative measurements were performed on RNA samples originating from 5 independent replicates. The quantification was performed with a LightCycler^® ^instrument using the LightCycler Fast Start DNA Master SYBR green I kit (Roche Diagnostics). Data were normalized using the ratio of the target cDNA concentration to that of the *glyceraldehyde 3-phosphate dehydrogenase *(*gapdh*) gene. The expression of this gene is not significantly influenced by the treatments and it is similar to the expression of the ribosomal protein L29 gene (data not shown). Primers were designed to amplify fragments with less than 300 bp; wpgrp1-For and wpgrp1-Rev generate a wpgrp1 fragment of 248 bp in length, and gapdh-For and gapdh-Rev generate a gapdh fragment of 277 bp in length (Table 1).

The PCR reactions were carried out in LightCycler 96-well plates in a final volume of 10 μl containing 2.5 μl of cDNA samples (diluted fivefold) and 7.5 μl of Light Cycler^® ^480 SYBR Green Master 1 mix, with 0.5 μl of 10 mM of each primer, 1.5 μl H2O and 5 μl of Mastermix. After 5 min at 95°C, the cycling conditions were as follows: 45 cycles at 95°C for 10 s, 56°C for 20 s and 72°C for 30 s. For product identification, a melting curve was constructed at the end of each PCR by heating for 30 s at 66°C and then increasing the temperature up to 95°C with increment rates of 0.11°C/s. Reactions were achieved by cooling at 40°C for 30 s.

The PCR efficiency (The efficienty (E) is calculated from the slope of the standard curve, which is obtained with different matrix quantities. E = 10^-1/slope^, in this study E was 97.7% for the *gapdh *and 94.4% for the *wpgrp1*) and, for the individual samples, the crossing point (Cp, the point at which amplified product is first visible in the data) and the concentration (conc.) of the *wpgrp1 *(or the *gapdh*) transcripts were determined. As the quantification relies on the PCR efficiency of each experiment, ratios were then normalized with the *gapdh*. The relative ratio for each sample was calculated according to the formula: (conc. *wpgrp1 *(sample)/conc. *gapdh *(sample)). Normalized data were analyzed using the one-way ANOVA and Tukey HSD post hoc test [24].

### Expression of recombinant Protein in *Escherichia coli *for antibody preparation

Total cDNA from larval bacteriomes of *Sitophilus zeamais *served as a template in PCR amplification for isolating the *wpgrp1 *cDNA.

The primers used were For-wpgrp1 cDNA and Rev-wpgrp1 cDNA (Table 1). The resulting DNA encoded 263 residues of the mature protein. The PCR product (789 pb) was cloned into a pTrc-His-Topo expression vector (Invitrogen) and the resulting vector was introduced into an *E. coli *TOP10 strain. The nucleotide sequence of the synthesized gene was checked by dideoxynucleotide sequencing and the recombinant plasmid was transformed into the *E. coli *BL21 strain. Expression of the recombinant protein Wpgrp1 flanked with 6 histidines at N-terminus was induced by 1 mM isopropyl-D-thiogalactopyranoside at an optical density of 0.6 at 30°C for 4 hours.

Bacterial lysates were prepared by sonication in buffer A, pH 8 containing 50 mM sodium phosphate, 300 mM NaCl and lysozyme to a final concentration of 1 mg/ml. The fusion protein bound to a Protino-Ni column (Macherey-nagel) was eluted with buffer A containing 250 mM imidazole after the column had been washed with buffer A. The eluted fraction was dialyzed against buffer containing 0.05 M Tris-HCl (pH 8.8), 1 mM EDTA and 10% glycerol. The protein was mixed with 2× SDS loading buffer and then, separated by electrophoresis in a 12.5% acrylamide gel. Next, the protein band was cut, N-terminal sequenced, and sliced into pieces for injection into rabbit to generate polyclonal Wpgrp1 antiserum at CovalAb Lyon (France), which was harvested 74 days after the initial inoculation.

### Western-blot analysis

Western blot analysis was performed using an enhanced chemiluminescence (ECL) western blotting analysis system (Amersham Biosciences, Piscataway, NJ, USA). The protein samples from the bacteriome were mixed with the sample buffer, boiled for 5 min, and loaded onto a 12.5% SDS-PAGE. The proteins were blotted onto a sheet of PVDF membrane (Amersham). After blotting, the membrane was blocked by incubation in a 3% gelatin solution, incubated with anti-wpgrp1 antiserum solution (1:500 v/v) at room temperature for 2 h and washed in TBST (100 mM Tris-HCl, pH 8,100 mM NaCl, 0.1% Tween20). For normalization, blots were probed with a β-tubulin antibody raised from the *Drosophila melanogaster *β-tubulin (tebu-bio, 1:500 v/v). The membrane was then incubated with 1:5000 (v/v) diluted anti-rabbit IgG-peroxydase antibody produced in goat (Sigma). After repeated washing, the membrane was incubated with ECL detection reagents (Amersham Biosciences) and exposed to a film.

## Results and Discussion

In this paper, we have first developed a new protocol to allow artificial rearing of weevils with access to the different stages of development and, secondly, we have demonstrated that RNAi technology can be applied to inhibit gene expression.

### Applying an artificial rearing methodology for weevil manipulation

Unlike *Tribolium*, weevil larvae are unable to feed on wheat flour or to survive outside cereal grains for behavioral and morphological reasons. Larvae require a compact texture that applies pressure on their back, which is required for an access to the food with their mandibles. To mimic this structure, we have set up an artificial rearing apparatus that consists of depositing weevil embryos and larvae between two layers of packed wheat flour (see Fig. 1). To access the later stages of the weevil life cycle (i.e. third and fourth larval stages), larvae were directly harvested from grain dissection. Early development is accessible using the embryo stages recovered from 2- to 4-hr layings on starch kernels. Embryos are collected once the females have laid by dissolving starch in water. They can either be manipulated or transferred to the GPF system to obtain the later developmental stages. Survival rates presented in Table 2 show that up to 34% of embryos succeeded in reaching the adult stage. When larvae are transferred and raised in the GPF, the yield is close to 100% (data not shown).

**Table 2:** Percentage of adult emergence of weevil embryos raised within GPF

	Number of embryos per experiment	% of adult emergence	Development time (days)*
Experiment 1	41	34.1	37
Experiment 2	38	36.8	38
Experiment 3	42	33.9	38
Average		33.9	37.7 ± 0.74

* The natural development time from the embryo to the adult of symbiotic weevils (at 27.5°C and 70 rh) is arround 30 days.

This system is of interest to applied and fundamental sciences as it allows easy access to different stages. Drugs and molecules of interest could be mixed with the flour and, thus, be tested directly on the insect variants.

### Testing RNAi-mediated gene silencing in *Sitophilus*

**Figure 2:**
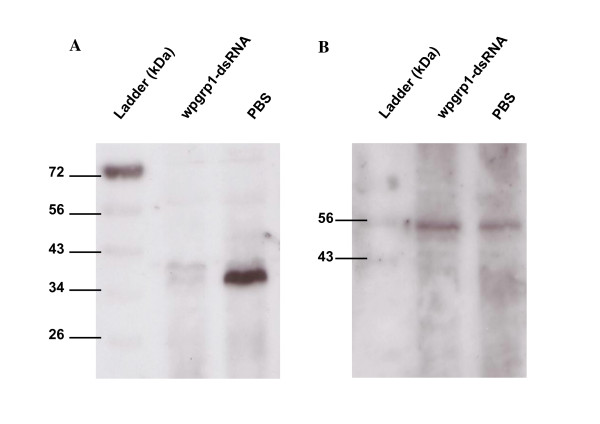
**Western blot analysis of Wpgrp1 protein steady-state levels in the bacteriome tissue four days after larval treatment**. Injection of gfp-dsRNA gave a similar result to the PBS-treatment (data not shown). A and B are blots probed with anti-Wpgrp1 and anti-tubulin antibodies, respectively.

RNAi was tested on *Sitophilus *insects with the *wpgrp1 *gene as a target. We recently identified the *wpgrp1 *gene by suppression subtractive hybridization while screening for genes, which expression is upregulated in the bacteriome tissue [14]. To investigate the *wpgrp1 *function in more detail, we have raised a Wpgrp1 polyclonal antibody and we have confirmed the presence of a high amount of wpgrp1 protein within the bacteriome by Western blot analysis (Fig. 2).

**Figure 3:**
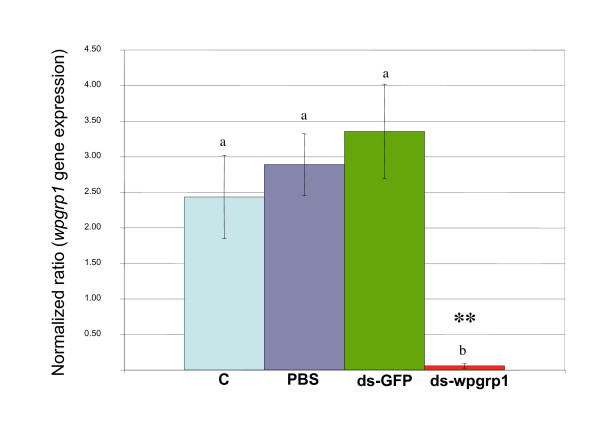
**Q-RT PCR analysis of *wpgrp1 *transcripts isolated from bacteriome tissues taken from fourth instar larvae four days after the treatments**. C, are untreated control larvae; PBS, ds-gfp and ds-wpgrp1 are larvae injected with PBS, gfp-dsRNA and wpgrp1-dsRNA, respectively. Data are normalized with the *gapdh *transcript levels, expressed as means of 5 independent repetitions and analyzed with one-way ANOVA and Tukey HSD post hoc test. The asterisks show significant differences (*p *< 0.05).

When third instar larvae were injected with sterile PBS or with *gfp*_dsRNA, the *wpgrp1 *transcript level in the bacteriome was within the same range as in the bacteriome of the uninjected control larvae. However, an injection of 200 ng of *wpgrp1*_dsRNA in the larval hemolymph leads to a 98% reduction in the target gene, compared to *gfp*_dsRNA control in the bacteriome *wpgrp1 *transcript levels (Fig. 3). Furthermore, this downregulation at the transcript level also affects the amount of Wpgrp1 protein in the bacteriome (Fig. 2). These data highlight the efficiency of RNAi gene silencing in *Sitophilus *insects and show that the RNAi technique can be successfully used to knockdown target genes in the bacteriome tissue.

## Conclusion

RNAi is a method by which dsRNA can be introduced directly into animals to trigger significant suppression of specific gene expression. In the light of our data, establishing the systemic RNAi pathway in this cereal weevil paves the way for more detailed comprehensive studies, on a molecularly level of weevil biology. The systemic RNAi pathway may also provide opportunities for developing species-specific, and, hence, ecologically friendly pest control methods. Genomic approaches have recently provided 10,000 unigenes, which will help to develop genome-wide RNAi applications addressing fundamental questions in cereal weevil physiology, development and gene regulation.

## Authors' contributions

AV established and performed GPF system and RNAi experiments, performed Q-RT PCR; CVM expressed the wpgr1 protein and performed Western blot analysis; AL contributed in the RNAi experiments; AH conceived the work; All the authors contributed to the manuscript writing.
